# Copper homeostasis and cuproptosis: implications for neurodegenerative diseases

**DOI:** 10.3389/fnagi.2025.1688554

**Published:** 2025-10-13

**Authors:** Feng Tao, Mengxuan Lin, Xiang Meng, Linghui Huang, Bifang Zhuo, Siyi Jiang, Shizhe Deng, Zhihong Meng, Jiangwei Shi

**Affiliations:** ^1^First Teaching Hospital of Tianjin University of Traditional Chinese Medicine, Tianjin, China; ^2^National Clinical Research Center for Chinese Medicine Acupuncture and Moxibustion, Tianjin, China; ^3^Shanghai University of Traditional Chinese Medicine, Shanghai, China; ^4^Northern Jiangsu People's Hospital, Yangzhou, China

**Keywords:** copper, cuproptosis, copper homeostasis, neurodegenerative diseases, cell death

## Abstract

Copper (Cu) is a vital trace element required for sustaining life and is involved in numerous critical metabolic processes within the body. Cuproptosis, a newly recognized type of Cu-dependent cell death, is mechanistically distinct from apoptosis, autophagy, pyroptosis, and ferroptosis. It is characterized by abnormal Cu accumulation and aberrant interactions with key enzymes of the tricarboxylic acid (TCA) cycle, which lead to protein aggregation, loss of iron–sulfur cluster proteins, and proteotoxic stress, ultimately leading to cell death. Recent studies have revealed that Cu dyshomeostasis and cuproptosis are intricately linked to the pathological progression of several neurodegenerative diseases, including Alzheimer’s disease (AD), Parkinson’s disease (PD), amyotrophic lateral sclerosis (ALS), Huntington’s disease (HD), Wilson’s disease (WD), and Menkes disease (MD). In this review, we systematically elucidate the systemic Cu metabolism, the molecular mechanisms of cuproptosis, and its intricate interplay with different neurodegenerative disorders. We also examined the relationship between cuproptosis and other types of cell death. Finally, we discuss therapeutic strategies targeting cuproptosis and Cu dyshomeostasis to combat neurodegenerative diseases and propose potential directions for future research.

## Graphical abstract

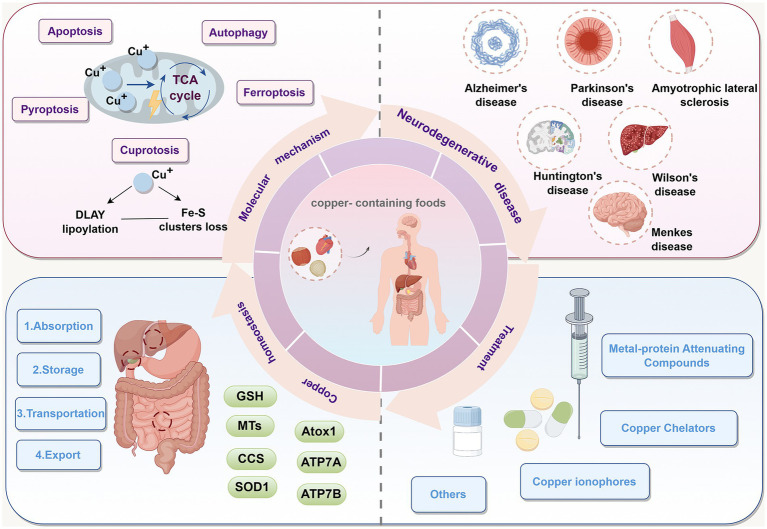

## 1 Introduction

Copper (Cu), being a crucial trace element, plays an important function in the human body (Festa and Thiele, 2011). It is a crucial cofactor for metalloenzymes like cytochrome c oxidase (CCO) and superoxide dismutase (Nývltová et al., 2022; Harris, 1992). Therefore, maintaining Cu homeostasis is essential for normal physiological function. When Cu accumulation exceeds the threshold maintained by homeostatic mechanisms, elevated Cu concentrations can exert toxic effects on cells and even lead to cell death (Linder, 2020). For a long time, the particular processes and various types of Cu-induced cell death were poorly understood. Only in 2022 did Tsvetkov et al. discover a new type of Cu-dependent regulated cell death, termed cuproptosis (Tsvetkov et al., 2022). It is different from the programmed cell death methods such as apoptosis, autophagy, ferroptosis and pyroptosis (Tsvetkov et al., 2022). The core mechanism of cuproptosis involves excessive Cu ions binding to lipoylated proteins within the mitochondrial tricarboxylic acid (TCA) cycle, such as dihydrolipoamide S-acetyltransferase (DLAT), leading to abnormal protein aggregation and loss of iron–sulfur (Fe-S) cluster, ultimately resulting in irreversible mitochondrial metabolic collapse and cell death (Tsvetkov et al., 2022; Tsvetkov et al., 2019).

The discovery of cuproptosis represents a major theoretical breakthrough in metal ion biology, reshaping the conceptual framework of the relationship between metal ion metabolism and cell fate and providing a novel perspective on the pathological mechanisms and therapeutic strategies for diseases. Cu is integral to the nervous system, participating in myelination, functioning as a vital element and structural component of various enzymes, and performs essential functions in electron and oxygen transport, protein modification, and neurotransmitter synthesis (Gaier et al., 2013). Growing evidence suggests that Cu dyshomeostasis and cuproptosis are intricately linked to the pathological progression of various neurodegenerative diseases, Alzheimer’s disease (AD), Parkinson’s disease (PD), amyotrophic lateral sclerosis (ALS), Huntington’s disease (HD), Wilson’s disease (WD), and Menkes disease (MD) (Cong et al., 2025; Bisaglia and Bubacco, 2020; Bandmann et al., 2015; Ahuja et al., 2015; Dexter et al., 1991). Understanding this newly discovered cell death pathway—cuproptosis—and its contribution to neurodegeneration is crucial for uncovering the fundamental nature of these diseases and the development of targeted therapeutic interventions.

This review summarizes the metabolic balance and physiological functions of Cu, explores the mechanisms of Cu-induced cell death, elucidates the role of Cu in neurodegenerative diseases, and highlights potential therapeutic strategies based on the regulation of Cu homeostasis and intervention in cuproptosis pathways. By integrating these emerging insights, this article aims to deepen our understanding of Cu-related pathological mechanisms in neurodegenerative diseases and to provide a theoretical basis and potential clinical direction for the development of novel neuroprotective strategies targeting Cu homeostasis or cuproptosis pathways.

## 2 Cu homeostasis and Cu metabolism

**Figure 1:**
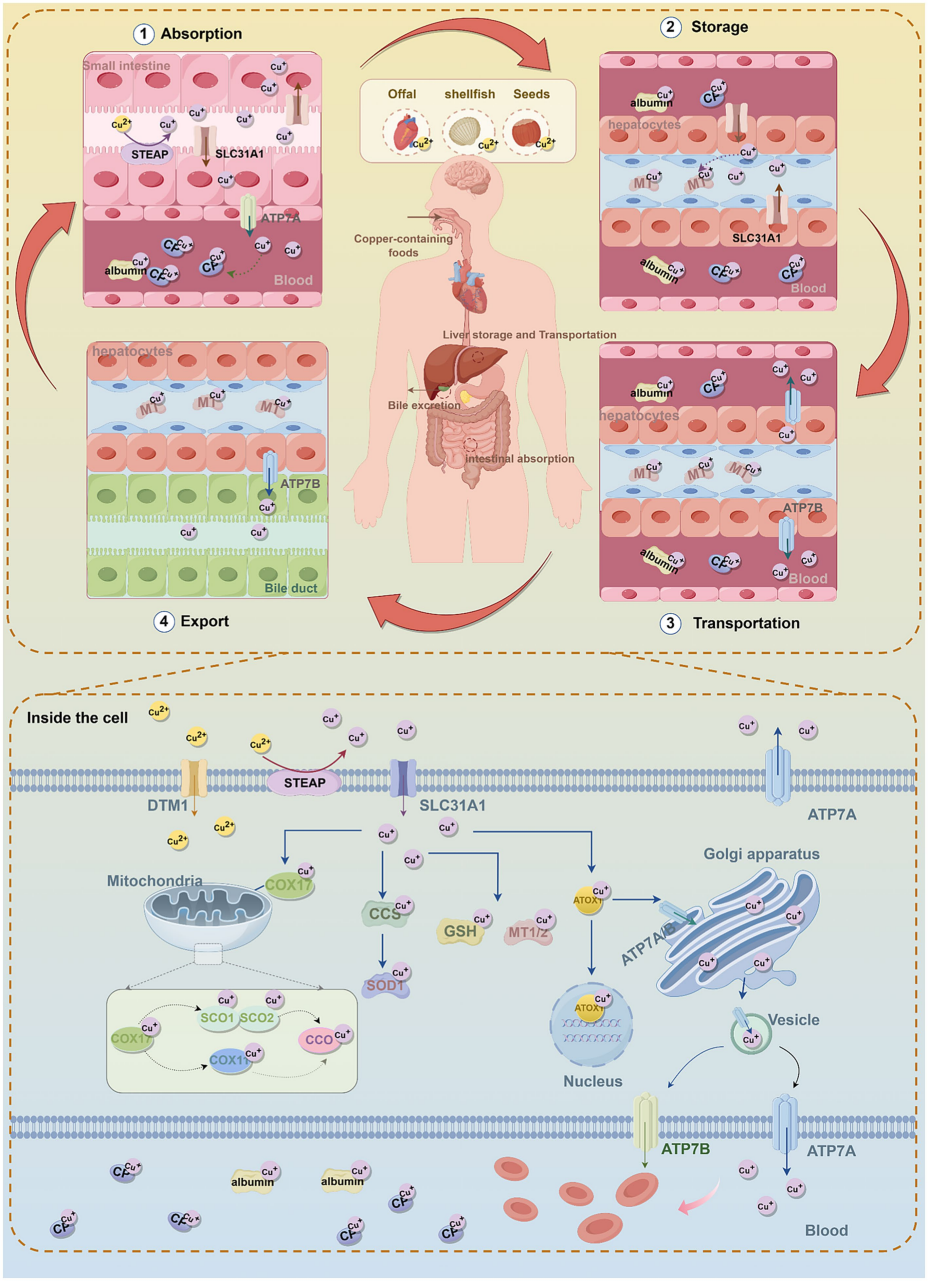
The molecular mechanisms of copper metabolism. In humans, dietary copper is mainly absorbed in the small intestine through the transporters SLC31A1 and DMT1, and Cu^2+^ is reduced to Cu^+^ by STEAP. In intestinal epithelial cells, Cu^+^ binds to copper chaperones and is transported to the basolateral side, where it is exported into the bloodstream via ATP7A. In the blood, Cu^+^ binds to copper-binding proteins, mainly CP. Copper is transported to the liver through the portal vein, and hepatocytes take up Cu^+^ from the bloodstream via SLC31A1. In hepatocytes, Cu^+^ can be stored in MT or re-enter the bloodstream through ATP7B to be distributed to other tissues. Excess copper is processed in the liver and excreted via bile, which is the main pathway of copper elimination. Inside cells, copper is delivered to and interacts with specific cytoplasmic copper chaperones, including COX17, CCS, and ATOX1, which direct copper to different cellular compartments, such as the mitochondrial electron transport chain, Golgi network, and nucleus. CCS delivers Cu^+^ to SOD1, protecting cells from oxidative stress. COX17 mainly transfers Cu^+^ to SCO1/2, and through the SCO1/2 or COX11 pathway, delivers Cu^+^ to CCO to activate the enzymes in the mitochondrial respiratory chain. ATOX1 directs Cu^+^ to ATP7A/B in the TGN. When copper is in excess, ATP7A/B translocate to vesicular compartments and fuse with the plasma membrane to export excess copper. In addition, ATOX1 participates in transporting copper to the nucleus, which is essential for the activation of various transcription factors. MT1/2 and GSH act as natural intracellular copper chelators, binding copper to store excess ions and prevent copper toxicity and cellular damage. Furthermore, the maintenance of intracellular copper homeostasis is achieved by ATP7A and ATP7B exporting excess copper ions into the bloodstream. Subsequently, copper is transported throughout the body via CP and albumin. SLC31A1, solute carrier family 31 member 1; STEAP, six-transmembrane epithelial antigen of the prostate; DMT1, divalent metal transporter 1; ATP7A/B, ATPase copper transporting alpha/beta; GSH, glutathione; MT1/2, metallothionein 1/2; CCS, superoxide dismutase; COX17/11, cytochrome c oxidase copper chaperone 17/11; CCO, Cytochrome c Oxidase; SCO1/2, sythesis of cytochrome oxidase 1/2; ATOX1, antioxidant 1 copper chaperone; SOD1, superoxide dismutase 1; CP, Cuproenzymes.

Humans primarily maintain Cu metabolic balance through dietary intake. Animal offal, shellfish, and seeds (including nuts and grains) are considered natural food sources with high Cu content (Linder and Hazegh-Azam, 1996) (Figure 1). Currently, the recommended daily Cu intake for adults range from 0.8 to 2.4 mg, and the total Cu concentration in the human body ranges from 100 to 200 mg (Bost et al., 2016). Although Cu is essential for growth and development, excessive intake can trigger the Fenton reaction (Cu^+^ + H_2_O_2_ → Cu^2+^ + • OH + OH^−^), leading to the generation of hydroxyl radicals (• OH), • OH is an important reactive oxygen species (ROS) that cause DNA, protein, and lipid damage, ultimately inducing cytotoxicity (Stohs and Bagchi, 1995; Shackelford et al., 2000). Therefore, Cu homeostasis in the body is tightly regulated by an intricate network of Cu-associated proteins, encompassing Cuproenzymes, Cu chaperones, and Cu transport protein (Lutsenko, 2010). These proteins collaboratively regulate Cu uptake, transport, storage, and excretion, thereby maintaining systemic Cu balance (Gao et al., 2023) (Figure 1).

Cu absorption primarily occurs in the stomach and the small intestine, with the duodenum being the principal site of uptake (Lönnerdal, 2008; Turnlund et al., 1998) (Figure 1). Upon ingestion, dietary Cu^2+^ must first be reduced to Cu^+^ by the six-transmembrane epithelial antigen of the prostate (STEAP) (Knutson, 2007) (Figure 1; Table 1). Subsequently, Cu^+^ is actively transported into intestinal epithelial cells via Cu transporter 1 (CTR1), also known as solute carrier family 31 member 1 (SLC31A1) (Nose et al., 2006) (Figure 1; Table 1). CTR1 is a trimeric channel protein located on the cell membrane, mainly responsible for transporting Cu from the extracellular space to the intracellular space (Lee et al., 2001; Larson et al., 2010). Evidence indicates that CTR1 levels are subject to negative feedback control by Cu concentration. Under Cu overload conditions, CTR1 expression is significantly downregulated to reduce Cu influx; conversely, during Cu deficiency, CTR1 expression adaptively increases to promote Cu absorption (Kuo et al., 2006). In addition, Cu can also enter cells via divalent metal transporter 1 (DMT1) and through passive diffusion (Lin et al., 2015) (Figure 1; Table 1).

**Table 1:** Cu homeostasis-related regulatory proteins.

Protein	Type	Function	Ref
STEAP	Metal reductase	Reduces oxidized Cu^2+^ to the absorbable Cu^+^ form, thereby facilitating Cu transmembrane transport.	Knutson (2007)
CTR1	Copper Transport Protein	Mediates transmembrane influx of Cu^+^ and serves as the principal channel protein for cellular Cu uptake.	Nose et al. (2006)
DMT1	Divalentmetal-ion transporter	Non-specific transport of Cu^2+^ involved in transmembrane Cu transport.	Lin et al. (2015)
Cp	Cuproenzymes	Circulating carrier and storage reservoir of Cu	Kirsipuu et al. (2020)
MT	Metallothionein	Chelation of Cu reduces its metal toxicity.	Gromadzka et al. (2020)
GSH	Cu chelator	Chelation of Cu reduces its metal toxicity.	Freedman et al. (1989)
ATOX1	Cu chaperone	Receives Cu imported via CTR1 and safely transports and conveys it to the copper-transporting ATPases (ATP7A/B) situated in the Golgi apparatus.	Hamza et al. (2003)
COX17	Cu chaperone	Receives Cu from the cytoplasm and transports it to the intermembrane space of the mitochondria, where it is transferred to other chaperones (e.g., COX11, SCO1/2) and ultimately incorporated into cytochrome c oxidase (CCO).	Prohaska (2008)
CCO	Cuproenzymes	Utilizing copper to catalyze redox reactions.	Nývltová et al. (2022)
ATP7A	Cu-transporting ATPases	Regulation of Cu Transport.	Palmgren and Nissen (2011)
ATP7B	Cu-transporting ATPases	Regulation of Cu Transport.	Palmgren and Nissen (2011)
CCS	Cu chaperone	Specifically receives cytosolic Cu^+^ and delivers it to activate SOD1.	Skopp et al. (2019)
SOD1	Cuproenzymes	Antioxidant damage protection.	Trist et al. (2021)

STEAP, six-transmembrane epithelial antigen of the prostate; CTR1, copper transporter 1; DMT1, divalent metal transporter 1; CP, ceruloplasmin; MT, metallothionein; GSH, glutathione; ATOX1, antioxidant 1 copper chaperone; COX17, cytochrome c oxidase copper chaperone 17; CCO, Cytochrome c Oxidase; ATP7A/B, ATPase copper transporting alpha/beta; CCS, superoxide dismutase; SOD1, superoxide dismutase 1.

Once inside the intestinal cells, Cu can be delivered to specific proteins or cellular compartments through various Cu chaperones (such as superoxide dismutase [CCS], antioxidant 1 Cu chaperone [ATOX1], Cytochrome c oxidase Cu chaperone 17 [COX17], etc.) to exert its functions (Chen et al., 2020). Cu homeostasis is maintained through the involvement of the aforementioned Cu chaperones (Figure 1; Table 1).

In the cytoplasm, the Cu chaperone CCS directly interacts with Cu and transports it to superoxide dismutase 1 (SOD1) (Skopp et al., 2019) (Figure 1; Table 1). SOD1 is a critical antioxidant enzyme responsible for scavenging intracellular ROS and maintaining redox homeostasis (Trist et al., 2021). The activity of SOD1 depends on three key post-translational modification steps: acquisition of Cu and zinc, formation of an intramolecular disulfide bond, and dimerization (Kawamata and Manfredi, 2010; Banks and Andersen, 2019). CCS is accountable for the targeted transport of Cu and the formation of disulfide bonds, directly regulating the maturation and functional activation of SOD1 and modulating intracellular oxidative stress (Skopp et al., 2019) (Figure 1; Table 1). CCS expression is subject to a negative feedback mechanism for maintaining Cu homeostasis: CCS expression increases under low intracellular Cu levels, whereas increased CCS protein inactivation occurs when cells transit from a Cu-deficient to a Cu-sufficient state (Tafuri et al., 2015).

Mitochondria are the primary organelles for Cu storage and utilization, playing a pivotal role in maintaining intracellular Cu homeostasis (Pierrel et al., 2007). Cytochrome c oxidase COX17, located in the mitochondrial intermembrane space, is a Cu-binding metallochaperone mainly responsible for transferring Cu to sythesis of cytochrome oxidase 1/2 (SCO1/2) (Prohaska, 2008; Voronova et al., 2007) (Figure 1; Table 1). SCO1/2 and cytochrome c oxidase Cu chaperone 11(COX11) are, respectively, responsible for incorporating Cu ions into the core subunits of CCO, COX2 (CuA site) and COX1 (CuB site), which is essential for the formation of an active enzyme (Banci et al., 2008; Horng et al., 2004) (Figure 1). Notably, CCO is an enzyme involved in the respiratory chain and redox reactions. In humans, CCO consists of two core subunits, COX1 and COX2, and plays a crucial role in regulating intracellular biochemical processes (Nývltová et al., 2022) (Figure 1; Table 1).

The Golgi apparatus serves as the central compartment for Cu homeostasis regulation (Hartwig et al., 2021). ATPase Cu-transporting alpha (ATP7A) and ATPase Cu-transporting beta (ATP7B) are the primary transporters responsible for exporting Cu from cells. Their localization and function are dynamically regulated and play a critical role in maintaining both cellular and systemic Cu balance (Palmgren and Nissen, 2011) (Figure 1; Table 1). Under physiological Cu levels, ATP7A/B are localized to the trans-Golgi network (TGN), where they pump Cu into the TGN lumen via the Cu chaperone ATOX1 (Hamza et al., 2003) (Figure 1; Table 1). In addition, ATOX1 can bind Cu and act as a transcription factor by associating with the promoters of specific genes (e.g., cyclin D1), thereby driving their expression and promoting cell proliferation (Itoh et al., 2008) (Figure 1; Table 1). When intracellular Cu levels are excessive, ATP7A and ATP7B can relocate to vesicular compartments and fuse with the plasma membrane to excrete surplus Cu, thus preventing Cu toxicity (Robinson and Winge, 2010). Notably, ATP7A is expressed in most tissues except the liver. In enterocytes of the small intestine, ATP7A mediates the transport of Cu into the bloodstream, facilitating its systemic distribution.

In the bloodstream, the concentration of Cu is typically maintained at a level of 0.7–1.4 μg/mL (Cartwright and Wintrobe, 1964), most Cu is bound to ceruloplasmin (Cp), while a smaller fraction is associated with albumin, transferrin, and free amino acids, and is transported to the liver via the portal venous system (Kirsipuu et al., 2020; Piacenza et al., 2021). In addition to the liver, circulating Cu can also be taken up by other tissues and organs, including the heart, skeletal muscle, and brain (Linder et al., 1998). The liver serves as the primary site for Cu storage and excretion, where excess Cu (The Cu content in the liver is approximately 15–55 μg/g of wet weight (Smallwood et al., 1968)) is exported from hepatocytes into bile in vesicular form through ATP7B (Aggett, 1999; Hernandez et al., 2008). Metallothionein1/2 (MT1/2) and glutathione (GSH) act as natural intracellular Cu chelators, binding Cu to sequester excess ions and thereby preventing Cu toxicity and cellular damage (Gromadzka et al., 2020; Freedman et al., 1989) (Figure 1; Table 1).

Overall, the maintenance of intracellular Cu homeostasis primarily relies on the regulation of Cuproenzymes, Cu chaperones, and Cu transport protein, while disruption of this balance can lead to metabolic disorders and even cell death (Asthana et al., 2014). This intricate equilibrium is essential for normal physiological processes and for preventing Cu dyshomeostasis-related diseases, such as various cancers and neurodegenerative disorders (Zhong et al., 2024; Yang et al., 2024; Huang et al., 2024).

## 3 Discovery and characteristics of cuproptosis

The research on Cu ion-dependent cell death mechanisms dates back to the 1980s, when Cu was first recognized for its biological capacity to trigger cell death (Halliwell and Gutteridge, 1984). However, due to technological limitations at the time, the underlying molecular mechanisms remained elusive for decades. Cu ionophores can transport Cu ions across the plasma membrane or mitochondrial membranes, and have played a pivotal role in the discovery of cuproptosis (Xie et al., 2023). Disulfiram (DSF), a drug used for the treatment of alcohol dependence, also functions as a Cu ionophore and has been reported to induce cell death (Cen et al., 2004), similar to another Cu ionophore, elesclomol (ES), which is likewise considered cytotoxic (Kirshner et al., 2008). In studies on the Cu ionophores ES and DSF, many researchers have investigated the mechanisms underlying the cell death induced by these agents, demonstrating that the death is caused by Cu itself rather than by the ionophores; however, the precise mechanism has not yet been clarified (Nagai et al., 2012).

**Figure 2:**
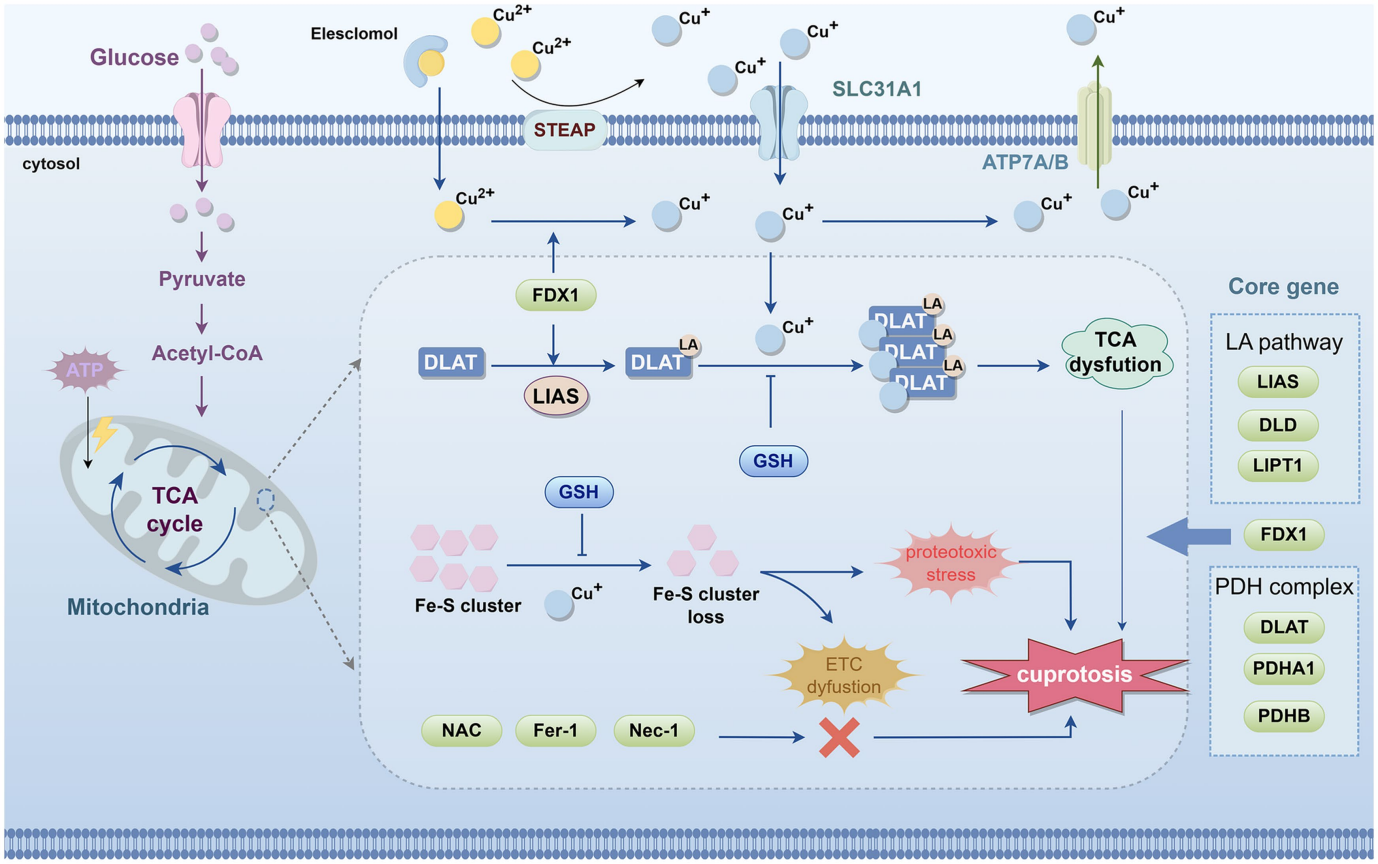
Schematic diagram of the process of cuproptosis. The occurrence of cuproptosis is associated with an increase in intracellular free Cu^+^ concentration, and extracellular copper is sequestered and transported into the cell by the copper ionophore elesclomol. FDX1/LIAS, as upstream regulators of protein lipoylation, enhance the lipoylation of TCA cycle enzymes such as DLAT. In mitochondria, FDX1 promotes the reduction of Cu^2+^ to Cu^+^. Subsequently, Cu^+^ binds to the lipoic acid thiol groups on DLAT, inducing abnormal oligomerization of DLAT and destabilizing Fe–S cluster proteins, leading to their loss. These molecular events collectively result in dysfunction of the TCA cycle and ETC, triggering acute proteotoxic stress and ultimately causing cell death. Notably, copper chelators such as GSH can suppress copper toxicity. However, NAC, ferrostatin-1, and necrostatin-1 do not alleviate copper-induced cell death. The core genes of cuproptosis include LIAS, DLD, and LIPT1 from the LA pathway; FDX1; and DLAT, PDHA1, and PDHB from the pyruvate dehydrogenase complex. FDX1, ferredoxin 1; DLAT, dihydrolipoamide S-acetyltransferase; TCA, tricarboxylic acid; ETC, electron transport chain; LA, Lipoic acid; LIAS, lipoic acid synthase; LIPT1, lipoyltransferase 1; DLD, dihydrolipoamide dehydrogenase; PDH, pyruvate dehydrogenase; DLAT, dihydrolipoamide S-acetyltransferase; PDHA1, pyruvate dehydrogenase E1 subunit *α*1; PDHB, pyruvate dehydrogenase E1 subunit β; GSH, glutathione; TTM, tetrathiomolybdate.

A revolutionary paradigm shift occurred in this field in 2022, when the research team led by Tsvetkov first elucidated the mechanism of Cu-induced cell death and formally designated it as “cuproptosis” (Tsvetkov et al., 2022), marking the beginning of a new era in research on Cu-induced cell death (Figure 2).

Tsvetkov et al. demonstrated through cell viability assays (e.g., MTT assay) that the Cu ionophore ES, in combination with Cu^2+^, elevates intracellular Cu levels and induces cell death, whereas ES alone exerts no such effect (Tsvetkov et al., 2022). Furthermore, by employing a range of established cell death inhibitors, they found that this form of cell death does not entail the cleavage or activation of the apoptotic marker caspase-3 (Tsvetkov et al., 2022). Notably, only Cu chelators, such as GSH, can rescue cells from this cytotoxicity (Tsvetkov et al., 2022). The ablation of essential apoptotic effectors BAX and BAK1, as well as the application of inhibitors aimed at many established cell death pathways— including ferroptosis inhibitors (ferrostatin-1), necroptosis inhibitors (necrostatin-1), and the ROS inhibitor N-acetylcysteine (NAC)—failed to avert this particular mode of cell death (Tsvetkov et al., 2022; Li et al., 2022). These data suggest that cuproptosis constitutes a unique form of cell death, separate from established cell death pathways (Tsvetkov et al., 2022).

Multiple studies have demonstrated that when intracellular Cu concentrations exceed the physiological threshold, they can cause mitochondrial damage and interfere with the function of key enzymes in the tricarboxylic acid (TCA) cycle (Arciello et al., 2005; Sheline and Choi, 2004). Tsvetkov et al. (2022), through a large-scale screening of 489 human cancer cell lines, found that cells highly dependent on mitochondrial respiration are more sensitive to Cu-induced cell death, whereas those relying predominantly on glycolysis are relatively resistant. Further investigations revealed that inhibition of electron transport chain (ETC) complexes or blockade of mitochondrial pyruvate uptake markedly attenuates Cu-induced cell death, indicating that mitochondria are the primary target of cuproptosis. In addition, cells treated with the ES-Cu complex exhibited a time-dependent dysregulation of TCA cycle–related metabolites, which represents a key mechanism driving the progression of cell death and further underscores the close association between cuproptosis and TCA cycle dysfunction (Tsvetkov et al., 2022).

Tsvetkov et al. performed a genome-wide CRISPR/Cas9 screen in sensitive cell lines (such as the human melanoma cell line SK-MEL-5), followed by genetic screening and single-gene knockout validation, and identified seven core genes that regulate cuproptosis. These include FDX1; components of the lipoic acid (LA) pathway—lipoic acid synthase (LIAS), lipoyltransferase 1 (LIPT1), and dihydrolipoamide dehydrogenase (DLD); and components of the pyruvate dehydrogenase (PDH) complex—dihydrolipoamide S-acetyltransferase (DLAT), pyruvate dehydrogenase E1 subunit α1 (PDHA1), and pyruvate dehydrogenase E1 subunit *β* (PDHB) (Figure 2) (Tsvetkov et al., 2022). Through protein immunoblotting and gene knockout experiments, FDX1 and LIAS have been identified as upstream regulators of protein lipoylation. Protein lipoylation is a highly conserved lysine post-translational modification, specifically present in four enzyme complexes involved in mitochondrial metabolism (Rowland et al., 2018; Solmonson and DeBerardinis, 2018). LIAS attaches lipoic acid to DLAT, which, as the E2 component of the PDH complex, catalyzes the conversion of pyruvate to acetyl-CoA in the TCA cycle (Rowland et al., 2018; Zhu et al., 2024). FDX1 is a reductase that reduces Cu^2+^ to Cu^+^ and has been confirmed as a direct target of ES (Tsvetkov et al., 2019). Subsequently, Cu^+^ binds to the lipoic acid thiol groups on DLAT, inducing abnormal oligomerization of DLAT and destabilizing iron–sulfur (Fe-S) cluster proteins, leading to their loss. These molecular events collectively cause dysfunction of the TCA cycle and ETC, triggering acute proteotoxic stress (Tsvetkov et al., 2022) (Figure 2).

In summary, as illustrated in Figure 2, cuproptosis can be described as follows: the Cu ionophore ES transports extracellular Cu^2+^ into the cell, where it is reduced to Cu^+^ by FDX1. Cu^+^ binds with high affinity to the lipoyl groups of mitochondrial lipoylated metabolic enzymes, such as DLAT, inducing protein misfolding, oligomerization, and aggregation, accompanied by the loss of Fe-S cluster proteins. This triggers severe mitochondrial proteotoxic stress, ultimately leading to cell death.

## 4 Cuproptosis and other forms of cell death

**Figure 3:**
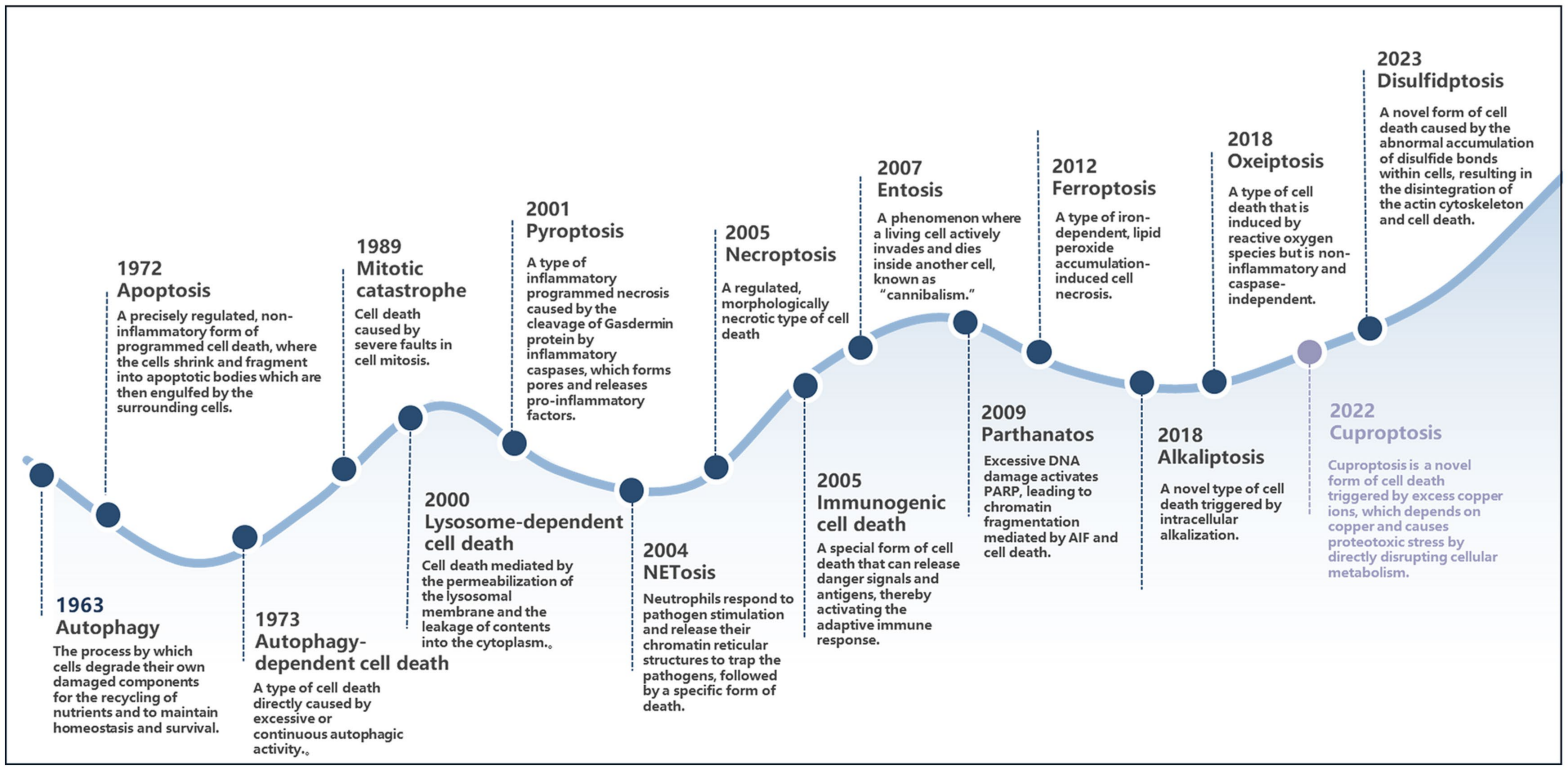
The timeline of the discovery of different forms of cell death.

Cell death is a critical process for maintaining homeostasis and supporting development in multicellular organisms (Boada-Romero et al., 2020). Among these, programmed cell death (PCD) refers to an active and orderly form of cell death controlled by genes under physiological or pathological conditions to preserve internal stability. PCD encompasses various forms, including autophagy, apoptosis, autophagy-dependent cell death, mitotic catastrophe, lysosome-dependent cell death, pyroptosis, NETosis, necroptosis, immunogenic cell death, entosis, parthanatos, ferroptosis, alkaliptosis, oxeiptosis, cuproptosis, and disulfidptosis (Hengartner, 2000; Cookson and Brennan, 2001; Brinkmann et al., 2004; Casares et al., 2005; Degterev et al., 2005; Overholtzer et al., 2007; David et al., 2009; Shen and Codogno, 2011; Vitale et al., 2011; Dixon et al., 2012; Aits and Jäättelä, 2013; Liu and Levine, 2015; Holze et al., 2018; Song et al., 2018; Tang et al., 2022; Liu et al., 2023). To provide a clearer overview of the progression of research on cell death, Figure 3 presents a timeline illustrating the discovery of these PCD models.

**Figure 4:**
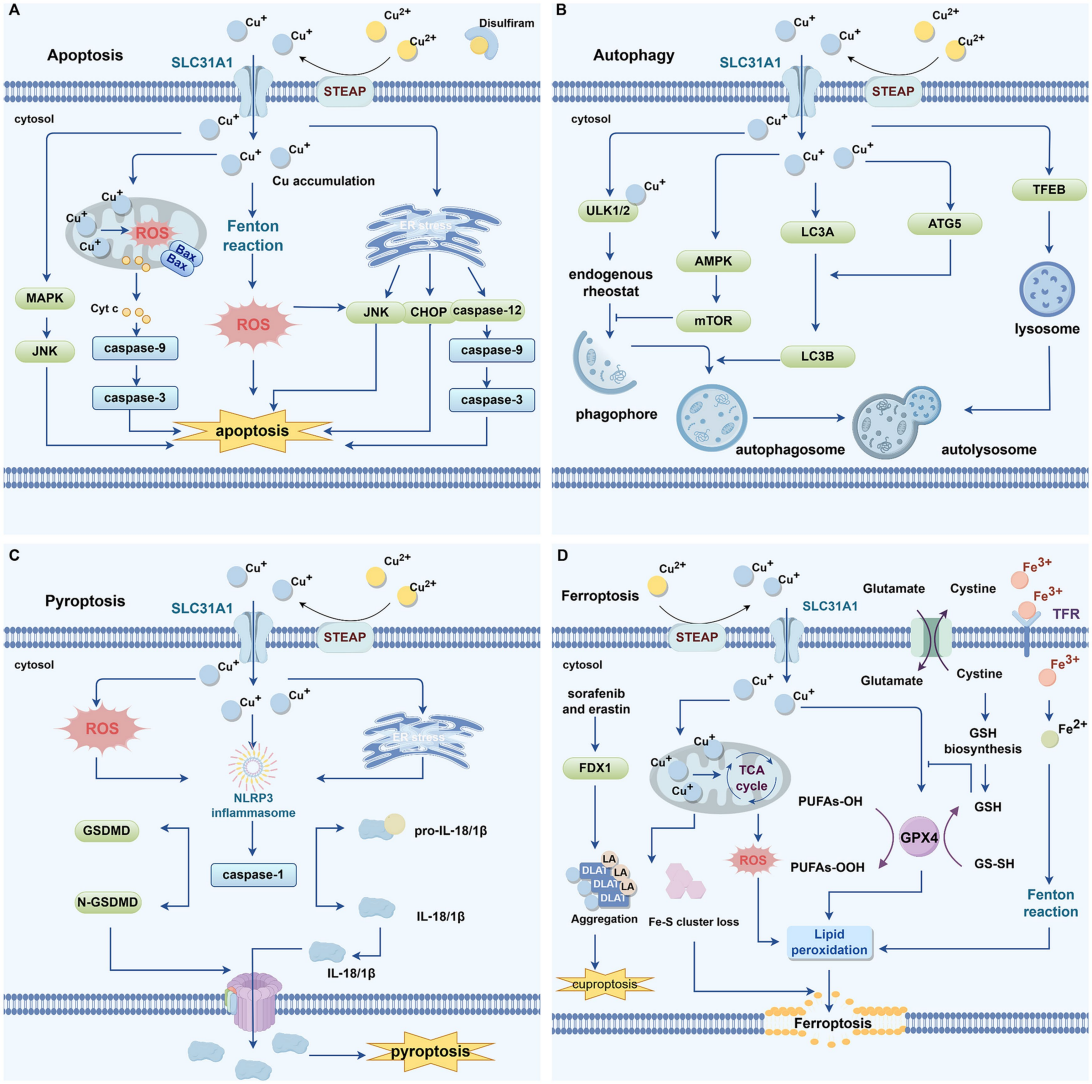
Cu and programmed cell death. **(A)** Excessive intracellular buildup of copper has been linked to apoptosis. High levels of copper can induce excessive ROS production through the Fenton reaction. Mitochondrial copper accumulation causes mitochondrial stress, leading to an increase in Bax, which promotes the release of Cyt c from mitochondria into the cytoplasm. Cyt c then associates with apoptotic caspase-9, resulting in caspase-3 activation and mediating apoptosis. Copper-induced endoplasmic reticulum stress can promote apoptosis via activation of the CHOP/JNK/caspase-12 signaling pathway. DSF-Cu can also induce apoptosis by activating the JNK signaling pathway. **(B)** Excess copper triggers apoptosis. Copper can directly regulate the activity of autophagy kinases ULK1 and ULK2, promoting phagophore assembly and subsequent autophagosome formation. Copper-activated AMPK inhibits mTOR activity, thereby facilitating phagophore formation. Copper markedly upregulates the expression of autophagy-related genes such as LC3A, LC3B, ATG-5, and Beclin-1, inducing autophagy. Copper-mediated upregulation of autophagy genes and activation of the transcription factor TFEB contribute to the formation of autophagosomes and autolysosomes, thereby further promoting autophagy. **(C)** Excess copper induces pyroptosis. Cu stimulates ROS production and ER stress, promoting the formation of the NLRP3 inflammasome, which activates caspase-1. Caspase-1 cleaves GSDMD to generate the N-terminal domain that forms membrane pores, thereby promoting pyroptosis. **(D)** Excess copper induces ferroptosis. Ferroptosis inducers, such as sorafenib and erastin, can promote copper-induced cell death by upregulating FDX1 protein levels, enhancing the aggregation of lipoylated proteins, and downregulating GSH. Excess Cu-mediated reduction of Fe-S cluster proteins further facilitate ferroptosis. Mitochondrial accumulation of copper generates ROS, which promote lipid peroxidation and induce ferroptosis. GPX4, a key antioxidant enzyme that eliminates lipid peroxides through the consumption of glutathione, binds to copper and undergoes autophagic degradation, thereby triggering ferroptosis. Both Fe and Cu can catalyze ROS generation through the Fenton reaction, and the rapid depletion of GSH mediated by these metals further impairs the cellular antioxidant capacity, leading to abnormal ROS accumulation. Excessive ROS drive lipid peroxidation, ultimately activating ferroptosis. ROS, reactive oxygen species; GPX4, glutathione Peroxidase 4; FDX1, ferredoxin 1; GSH, glutathione; ER, endoplasmic reticulum; Cyt c, cytochrome c; MAPK, mitogen-activated protein kinase; JNK, Jun N-terminal kinase; ULK1/2, Unc-51-like autophagy activating kinase 1/2; mTOR, mechanistic target of rapamycin kinase; AMPK, AMP-activated protein kinase; ATG5, autophagy related 5; NLRP3, NOD-like receptor family pyrin domain-containing 3; GSDMD, gasdermin D.

Moreover, although Cu ionophores in complex with Cu constitute the primary pathway for inducing cuproptosis, intracellular Cu overload can also trigger other forms of PCD, including apoptosis, autophagy, pyroptosis, and ferroptosis (Zhang et al., 2024) (Figure 4). This suggests a molecular crosstalk between cuproptosis and other PCD pathways. A deeper understanding of Cu’s role across multiple modes of cell death is crucial for the development of more precise and effective cuproptosis-targeted therapies.

### 4.1 Cu and apoptosis

Excessive intracellular buildup of Cu has been linked to apoptosis (Figure 4A) (Ostrakhovitch and Cherian, 2005). It is well established that ROS and oxidative stress serve as potent inducers of apoptosis (Maiuri et al., 2007). Increased intracellular ROS can induce apoptosis by various mechanisms, including endoplasmic reticulum (ER) stress, mitochondrial impairment, and activation of death receptors (Liu et al., 2021). Studies have demonstrated that Cu can induce oxidative damage (Gaetke and Chow, 2003; Rakshit et al., 2018; Shimada et al., 2018), and high levels of Cu can lead to excessive production of ROS via the Fenton reaction (Jomova and Valko, 2011). Kawakami and colleagues reported that high Cu exposure induces apoptosis in PC12 cells by upregulating the expression of BAX, caspase-3, cytochrome c (Cyt c), and caspase-9 (Kawakami et al., 2008). Wu et al. demonstrated that CuSO₄-induced ER stress promotes apoptosis in mouse hepatocytes through activation of the CHOP, Jun N-terminal kinase (JNK), and caspase-12 signaling pathways (Wu et al., 2020). In osteosarcoma cells, DSF–Cu induces tumor cell apoptosis via activation of the JNK signaling pathway (Guo et al., 2021; Xu et al., 2020). These findings suggest that ROS may serve as a critical link between cuproptosis and apoptosis.

### 4.2 Cu and autophagy

Cu has been reported as a critical metal ion involved in the regulation of autophagy (Figure 4B) (Wan et al., 2020). Research has shown that Cu can directly regulate the activity of autophagy kinases Unc-51-like autophagy activating kinase 1(ULK1) and Unc-51-like autophagy activating kinase 2(ULK2), functioning as an endogenous rheostat to control the autophagic pathway (Tsang et al., 2020). A decline in intracellular ATP levels leads to an elevated AMP/ATP ratio, which activates AMP-activated protein kinase (AMPK) (Klionsky et al., 2016). Activated AMPK inhibits the activity of mTOR complex 1 (mTORC1), resulting in enhanced autophagy and facilitating the recycling of cellular materials and energy (Klionsky et al., 2016). Liao et al. noted that elevated Cu levels stimulate autophagy in the renal tissues of broiler chickens through the activation of the AMPK-mTOR signaling pathway (Liao et al., 2020). Other studies have demonstrated that Cu overload markedly enhances the expression of autophagy-related genes, such as LC3A, LC3B, autophagy related 5(ATG5), and Beclin-1, suggesting that surplus Cu can trigger autophagy in rat kidneys (Wan et al., 2020). Tang et al. reported that treatment with CuSO₄ markedly upregulated the expression of autophagy-related genes and proteins, including ULK1 and ATG16L1, in hepatocytes harboring the ATP7B R778L mutation (Tang et al., 2023). Moreover, Cu overload enhances autophagy levels via the Cu–mTORC1–TFEB signaling pathway, with TFEB functioning as a key regulator of lysosomal biogenesis (Polishchuk et al., 2019).

### 4.3 Cu and pyroptosis

Cu has the ability to affect pyroptosis (Figure 4C) (Liao et al., 2019). The research found that excessive Cu in liver cells can activate the expression of genes related to pyroptosis (such as Caspase-1, NOD-like receptor family pyrin domain-containing 3[NLRP3], IL-1β and IL-18) (Liao et al., 2019). NAC (a ROS scavenger) can inhibit this behavior, indicating that the ROS produced by Cu-induced action may be the main mediator of pyroptosis (Liao et al., 2019). Additionally, Liao et al. reported that in small intestinal epithelial cells, the expression levels of genes linked to pyroptosis—including NLRP3, gasdermin D (GSDMD), caspase-1, IL-18, and IL-1β—are significantly upregulated in response to elevated Cu levels, suggesting that Cu can induce pyroptosis in jejunal epithelial cells (Liao et al., 2022). This process is likely mediated via endoplasmic ER stress, as treatment with ER stress inhibitors reduces Cu-induced pyroptosis in intestinal epithelial cells (Liao et al., 2022).

### 4.4 Cu and ferroptosis

There exists a significant interplay between cuproptosis and ferroptosis (Figure 4D). GSH serves as a critical nexus, capable of chelating both Fe and Cu ions to mitigate metal-induced toxicity (Jomova and Valko, 2011). Ferroptosis inducers like sorafenib and erastin have been shown by Wang et al. to enhance cuproptosis in primary hepatocellular carcinoma cells, primarily by inhibiting FDX1 degradation, promoting protein lipoylation, and reducing GSH synthesis (Wang et al., 2023). Previous studies have shown that depletion of Fe–S cluster proteins can trigger ferroptosis (Alvarez et al., 2017). Glutathione Peroxidase 4 (GPX4), a key antioxidant enzyme that specifically eliminates lipid peroxides through GSH consumption, undergoes inactivation to directly cause lipid peroxide accumulation and initiate ferroptosis (Yang et al., 2014). GSH also acts as an essential cofactor for GPX4, in Pancreatic ductal adenocarcinoma (PDAC) cells, Cu binds to GPX4 and facilitates its autophagic degradation, thereby inducing ferroptosis (Xue et al., 2023). Mitochondrial metabolism represents another critical intersection between cuproptosis and ferroptosis. Studies have shown that DSF-Cu or ES-Cu can disrupt mitochondrial homeostasis, enhance lipid peroxidation, and ultimately lead to ferroptosis (Gao et al., 2021; Ren et al., 2021). Moreover, ROS are key mediators in the crosstalk between ferroptosis and cuproptosis (Jiang et al., 2021). Both Fe and Cu can catalyze ROS generation through Fenton reactions, and metal-induced rapid depletion of GSH further compromises cellular antioxidant defenses, resulting in excessive ROS accumulation (Jiang et al., 2021). Elevated ROS levels drive lipid peroxidation, eventually triggering ferroptosis (Jiang et al., 2021). Although Cu toxicity in ES-Cu-induced cuproptosis is not primarily caused by ROS production, excessive Cu-induced ROS formation and GSH depletion may still precipitate ferroptosis.

## 5 Cu and neurodegenerative diseases

### 5.1 Mechanisms of Cu-induced neuronal degeneration

**Figure 5:**
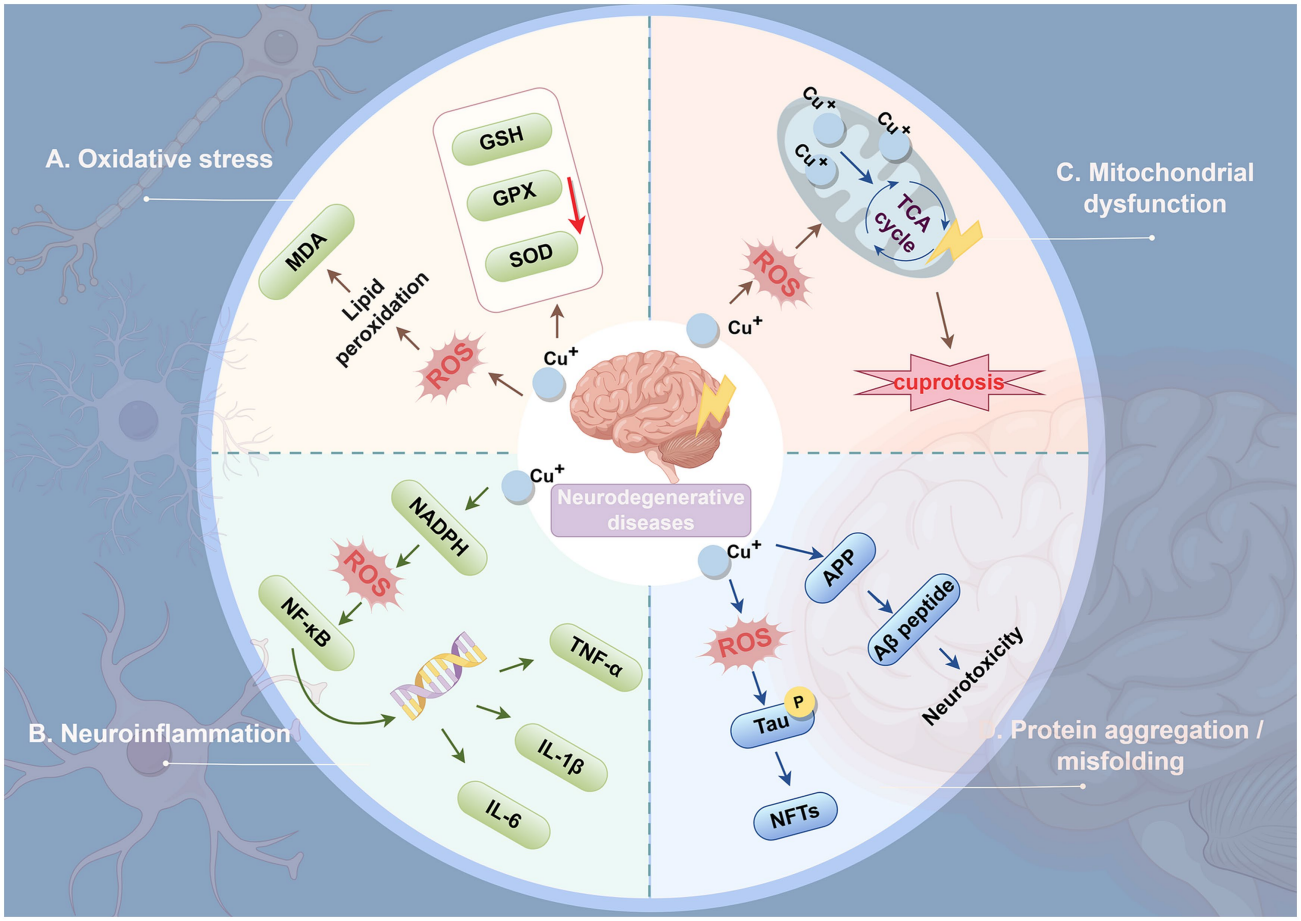
Mechanisms of Cu-induced neuronal degeneration. **(A)** Oxidative stress. Excess copper increases the production of ROS and promotes oxidative stress. Elevated ROS levels induce lipid peroxidation in the neuronal cell membrane, leading to the production of MDA, a cytotoxic compound. ROS also impair the ability of cells to synthesize antioxidant enzymes, further aggravating oxidative stress. **(B)** Neuroinflammation. Copper homeostasis abnormalities within neurons trigger ROS production. Consequently, the NF-κB signaling pathway becomes activated, thereby initiating the inflammatory cascade response. NF-κB promotes the transcription of inflammatory genes and the production of pro-inflammatory cytokines such as TNF-α, IL-1, IL-6, and IL-12. This leads to neurotoxicity and accelerates neurodegeneration by exacerbating redox cycling and inflammation. **(C)** Mitochondrial dysfunction. Elevated ROS levels may further impair mitochondrial integrity, forming a feedback loop that exacerbates ROS production. Cu markedly enhances mitochondrial ROS generation, subsequently impairing TCA cycle activity. **(D)** Protein aggregation/misfolding. Cu induces tau protein phosphorylation, and its activation leads to the formation of NFTs and subsequent neurodegeneration. Excessive copper in the brain triggers Aβ peptide aggregation and deposits on neurons, resulting in neurotoxicity. ROS, reactive oxygen species; MDA, malondialdehyde; NADPH, nicotinamide adenine dinucleotide phosphate; NF-κB: Nuclear factor-kappa B; GSH, glutathione; GPX, Glutathione peroxidase; SOD, superoxide dismutase; TCA, tricarboxylic acid; TNF-α: tumor necrosis factor alpha; IL-1β, Interleukin-1 beta; IL-6, Interleukin-6; APP, Amyloid precursor protein; Aβ, Amyloid-beta; NFTs, neurofibrillary tangles.

Imbalance of Cu homeostasis adversely affects neuronal function, triggering oxidative stress, neuroinflammation, mitochondrial dysfunction, and protein aggregation/misfolding, ultimately leading to the development of neurodegenerative diseases (Figure 5).

#### 5.1.1 Oxidative stress

Oxidative stress is a key factor in the pathogenesis of neurodegenerative diseases (Figure 5) (Roy et al., 2023). During aerobic metabolism, cells generate ROS; when ROS levels exceed the capacity of intracellular antioxidant systems, oxidative stress ensues. This condition causes widespread oxidative damage to biomolecules and disrupts signaling pathways, ultimately leading to cell death (Gorrini et al., 2013). Cu, as a redox-active transition metal, contributes to ROS generation (Vo et al., 2024). Cu exposure has been shown to progressively suppress the expression of SOD, GPX, and GSH peroxidase in the brain tissue of C57BL/6 J mice, while increasing malondialdehyde (MDA) levels, indicating that Cu induces lipid peroxidation and damage (Zhang et al., 2023). In astrocytes, Cu toxicity is associated with oxidative stress, as treatment with antioxidants mitigates Cu-induced cell death, demonstrating that oxidative stress mediates Cu-induced astrocyte cytotoxicity (Gale et al., 2023). Furthermore, in a human SH-SY5Y/astrocyte co-culture model, Cu pyrithione induces cytotoxicity at approximately 400 nM, inhibiting neurite outgrowth; at around 200 nM, it causes neurotoxicity and downregulates genes involved in neuronal development and maturation. These findings indicate that oxidative stress is the primary mechanism underlying Cu pyrithione-induced cytotoxicity in co-cultured cells (Oh and Kim, 2023).

#### 5.1.2 Neuroinflammation

As mentioned above, Cu exposure increases ROS production, which induces chronic oxidative stress in the brain and may further lead to neuronal dysfunction and neuroinflammation (Figure 5) (Patwa and Flora, 2024). Nuclear factor-kappa B (NF-κB) is a key transcription factor that regulates the expression of pro-inflammatory genes and serves as a principal regulator of the synthesis of major pro-inflammatory cytokines, including TNF-*α*, IL-1β, and IL-6 (Teleanu et al., 2022). Studies have shown that Cu can enhance ROS activity in vascular endothelial cells, thereby activating downstream signaling molecules such as IκB kinase (IKK). This process accelerates the translocation of NF-κB to the nucleus and triggers transcription of pro-inflammatory cytokines (Tsai et al., 2002).

#### 5.1.3 Mitochondrial dysfunction

Mitochondria are central targets of Cu-induced neurotoxicity, and their dysfunction represents a pivotal event leading to energy failure, oxidative stress bursts, and the eventual activation of cell death pathways (Figure 5) (Liao et al., 2020). Studies in SH-SY5Y human neuroblastoma cells treated with CuSO₄ (50–300 μM) demonstrated that Cu exposure markedly increases ROS production and decreases the activity of PDH within the TCA cycle (Arciello et al., 2005). In mixed cultures of neurons and glial cells, Cu exposure causes a decline in mitochondrial membrane potential and suppresses the activity of mitochondrial PDH and *α*-ketoglutarate dehydrogenase (KGDH), whereas treatment with antioxidants mitigates this Cu-induced enzymatic inhibition and subsequent cell death (Sheline and Choi, 2004). Furthermore, the recently identified form of Cu-induced cell death, cuproptosis, operates via mechanisms distinct from other forms of programmed cell death such as apoptosis, ferroptosis, or necroptosis, and is characterized by abnormal accumulation of Cu ions in the mitochondrial matrix, oligomerization of lipoylated mitochondrial proteins, and loss of Fe-S clusters (Tsvetkov et al., 2022).

#### 5.1.4 Protein aggregation/misfolding

A large number of proteins *in vivo* must fold into specific conformations to perform their biological functions. Proper protein folding and structural integrity are essential for maintaining normal neuronal function and underlie processes such as learning and memory (Cozachenco et al., 2023). However, protein misfolding or aggregation disrupts function and contributes to various neurodegenerative diseases (Figure 5) (Lonsdale, 2019). Excess Cu may induce oxidative stress, leading to hyperphosphorylation of Tau protein. Hyperphosphorylated Tau aggregates to form neurofibrillary tangles (NFTs), ultimately triggering neurodegeneration (Zubčić et al., 2020). Amyloid-beta (A*β*) peptides, derived from the enzymatic cleavage of amyloid precursor protein (APP), have been shown to aggregate in the brain under conditions of Cu overload, precipitating on neurons and causing neurotoxicity (Huang et al., 1999). Moreover, Cu can bind to various neuronal proteins, including α-synuclein, Tau, SOD1, and mutant huntingtin (mHTT), promoting their aggregation in specific brain regions. Deposition of these aggregates in neurons can ultimately induce neurotoxicity, with oxidative damage serving as a key contributing mechanism. These protein aggregates are associated with multiple neurodegenerative diseases, including AD, PD, ALS, and HD (Bandmann et al., 2015).

### 5.2 Dysregulation of Cu homeostasis and cuproptosis in disease

#### 5.2.1 Alzheimer’s disease

AD is a complex neurodegenerative condition marked by basic pathological features, including the accumulation of amyloid plaques and NFTs formed of hyperphosphorylated tau protein (Scheltens et al., 2021). Amyloid plaques are primarily composed of Aβ. The precursor of Aβ is the APP, which is processed by β-secretase and *γ*-secretase to produce Aβ (Delport and Hewer, 2022).

Disrupted Cu homeostasis has been implicated in cognitive impairment in patients with Alzheimer’s disease and may contribute to disease progression (Squitti et al., 2019). Studies have reported that serum Cu levels are significantly elevated in AD patients compared with healthy controls (Bucossi et al., 2011; Squitti et al., 2013). Previous research has shown a negative correlation between free Cu levels and cognitive performance, with higher Cu concentrations associated with poorer cognitive outcomes and progressive decline over time (Squitti et al., 2006; Squitti et al., 2009). Furthermore, Cu may impair synaptic plasticity by mediating cuproptosis and inhibiting the cAMP response element-binding protein (CREB)/brain-derived neurotrophic factor (BDNF) signaling pathway, ultimately leading to cognitive deficits in mice (Zhang et al., 2023).

Cu can interact with key pathological factors in AD, including APP, Aβ, and tau. The APP contains two Cu-binding domains: one located in the N-terminus and another within the Aβ sequence (Simons et al., 2002). The Cu^2+^ reductase activity associated with these domains, particularly the N-terminus, can promote the generation of ROS, thereby exacerbating Cu-induced neurotoxicity (Multhaup et al., 1996). APP knockout mice exhibit elevated Cu levels in their cerebral cortex relative to wild-type mice, suggesting that APP regulates Cu homeostasis (White et al., 1999). Aβ has a strong binding affinity for Cu^2+^, and among all subtypes, Aβ42 exhibits the strongest binding capacity (Jaragh-Alhadad and Falahati, 2022). Aβ42 can form a Cu^2+^-Aβ42 complex with Cu^2+^, which possesses strong reductive properties. This complex can exacerbate oxidative stress and contribute to the progression of AD pathology (Jaragh-Alhadad and Falahati, 2022). Furthermore, studies using the triple transgenic (3xTg-AD) mouse model have found that chronic Cu exposure induces an increase in tau protein phosphorylation, which can promote or exacerbate the progression of AD (Crouch et al., 2009).

Cu may also contribute to neuroinflammation in AD. Previous studies have indicated that Cu plays a critical role in maintaining microglial homeostasis (Zucconi et al., 2007). It has been reported that low-dose Cu exposure induces upregulation of APP, leading to microglial activation and TNF-*α*-mediated signaling, thereby promoting microglia-mediated neurotoxicity toward neighboring neurons (Lu et al., 2009). Chronic Cu exposure disrupts the homeostatic phenotype of microglia and exacerbates neuroinflammation (Lim et al., 2020).

Cuproptosis-associated genes are implicated in the pathological mechanisms of AD (Li et al., 2025). A study analyzed the expression profiles of Cu death regulators in the brains of AD patients and healthy individuals and found two distinct clusters of Cu death-related molecules in AD patients, highlighting its critical role in AD (Lai et al., 2022). In addition, another study screened four of the seven Cu death-related genes (including IFI30, PLA1A, ALOX5AP, and A4GALT) upregulation in AD through differential gene expression and weighted gene co-expression network analysis, and used these to construct a diagnostic model (Zhang et al., 2022). The study also investigated the correlation between immune cell infiltration in samples and these genes, further proving that Cu death-related genes play an important role in the pathological mechanism of AD (Zhang et al., 2022). PDHA1 is the core structure of the PDH complex and contains the active site of PDH. Studies have found that in AD mice model (APP-PS1 mice), the expression of the PDHA1 gene is significantly upregulated in the prefrontal cortex (PFC) and may play a key role in AD progression by potentially indirectly affecting APP processing. Therefore, it is considered a promising novel therapeutic target (Yang et al., 2020). LIAS is the main enzyme involved in LA biosynthesis, and its downregulation can lead to a decrease in alpha lipoic acid (ALA) biosynthesis (Li et al., 2025). ALA has been reported to exert beneficial effects in neurodegenerative diseases such as AD, improving cognitive performance in Tg2576 mice and protecting mitochondria from oxidative stress (Quinn et al., 2007).

#### 5.2.2 Parkinson’s disease

PD is a chronic, progressive neurodegenerative disorder characterized by the degeneration and demise of dopaminergic neurons in the substantia nigra and the presence of Lewy bodies (inclusions formed by abnormal aggregation of *α*-synuclein [α-Syn]) (Dickson, 2012). α-Syn, a small peripheral membrane protein specifically localized at the presynaptic terminals of neurons, plays a central role in the pathogenesis of PD (Liu et al., 2021).

Cu plays a role in the abnormal aggregation of α-Syn (Choi et al., 2018). Modified Cu levels and the enhanced production of α-Syn–Cu complexes may significantly influence ROS generation in the pathophysiology of PD (Li and Kerman, 2019). Studies have shown that Cu^2+^ binds to α-Syn to form a complex with dopamine oxidase-like activity, which catalyzes the generation of ROS via Cu^2+^/Cu^+^ redox cycling in the presence of biological reductants (Calvo et al., 2020; Carboni and Lingor, 2015). Moreover, the redox activity of the α-Syn–Cu complex may exacerbate cellular oxidative stress, leading to tyrosine cross-linking and oxidation of the neurotransmitter dopamine (Wittung-Stafshede, 2022). Given the high vulnerability of dopaminergic neurons to degeneration and death in PD, these findings suggest that increased deposition of α-Syn–Cu complexes due to Cu metabolism disorder is an important pathological hallmark of PD (Wittung-Stafshede, 2022).

Dysregulation or dysfunction of dopaminergic neurons is closely associated with PD (Post and Sulzer, 2021). Interestingly, excessive exposure to Cu can enable its passage across the blood–brain barrier, leading to degeneration of dopaminergic neurons (Raj et al., 2021). Recent studies have shown that Cu exposure can activate microglia to secrete proinflammatory mediators, inducing pyroptosis in dopaminergic neurons (Zhou et al., 2022). This process is linked to premature activation of the ROS/NF-κB signaling pathway and the resultant dysfunction of mitophagy in microglia derived from C57BL/6 J mice (Zhou et al., 2022).

Zhao et al. identified three cuproptosis-related genes—ATP7A, SLC31A1, and DBT—as participants in the immune processes of PD, providing important insights into the physiological and pathological roles of Cu toxicity in PD (Zhao et al., 2022). Additionally, Zhang et al. analyzed the expression profiles of cuproptosis-related genesin PD across multiple GEO datasets and identified KIAA0319, AGTR1, and SLC18A2 as core genes involved in PD pathogenesis. They also constructed a predictive model to assess PD risk, aiming to provide novel insights for therapeutic strategies in PD (Zhang et al., 2023). GSH chelates intracellular Cu to block its toxicity (Tsvetkov et al., 2022). When intracellular copper excessively accumulates due to insufficient GSH, it interferes with Fe-S cluster proteins, leading to the downregulation of Fe-S proteins that mediate cytotoxic stress and cell death (Tsvetkov et al., 2022). The GSH fluorescent probe R13 has revealed a reduction of GSH levels in the brains of PD mouse models (Zhang et al., 2023). Caffeine and uric acid, which are purine derivatives, have neuroprotective activity and are negatively correlated with the incidence of AD and PD (Lindsay et al., 2002; Ascherio et al., 2001). Paraxanthine, the major metabolite of caffeine, has been shown to elevate intracellular cysteine levels, thereby promoting GSH synthesis and exerting protective effects on neuronal cells (Matsumura et al., 2023). These lines of evidence further support the association between cuproptosis and the pathogenesis of PD.

#### 5.2.3 Amyotrophic lateral sclerosis

ALS is a progressive and incurable neurodegenerative disease that predominantly impacts upper and lower motor neurons, leading to damage to the muscles of the trunk, limbs, and craniofacial regions innervated by these neurons (Feldman et al., 2022).

Approximately 90% of amyotrophic lateral sclerosis (ALS) cases are sporadic (SALS), while 10% are familial (FALS). Several genes have been implicated in ALS, including TARDBP and C9ORF72; however, mutations in the SOD1 gene remain the most extensively studied to date (Abati et al., 2020; DeJesus-Hernandez et al., 2011; Sreedharan et al., 2008). More than 100 distinct SOD1 mutations account for approximately 20% of familial ALS cases, and SOD1 mutations are also present in sporadic cases of the disease (Rosen et al., 1993). SOD1 is a major intracellular Cu-binding protein localized in both the mitochondrial intermembrane space and the cytosol, requiring Cu and zinc as cofactors to regulate intracellular Cu homeostasis (Pansarasa et al., 2018). When SOD1 forms disulfide bonds prior to metal loading, the Cu chaperone for SOD1 (CCS) can no longer recognize or activate SOD1, thereby preventing Cu delivery and disulfide bond maturation (Boyd et al., 2020). In ALS, the interaction between CCS and mutant SOD1 is disrupted (Gil-Bea et al., 2017). Studies in ALS model mice (SOD1-G93A transgenic mice) have shown that metal deficiency at the Cu-binding site of mutant SOD1 represents one of the earliest pathological features of SOD1-associated ALS (SOD1-ALS) (Tokuda et al., 2018).

Bakkar et al. identified an M1311V mutation in ATP7A from ALS patients, which leads to altered subcellular localization and impaired function of ATP7A, thereby disrupting intracellular Cu transport and causing abnormal Cu accumulation (Bakkar et al., 2021). Moreover, relevant studies have shown that ATP7B expression is downregulated in the spinal cord of ALS model mice (SOD1-G93A transgenic mice) (Olsen et al., 2001). These results further substantiate the involvement of Cu homeostasis dysregulation as a pathogenic mechanism in ALS. Metallothioneins (MTs) are Cu- and zinc-binding proteins that play a crucial role in maintaining intracellular Cu homeostasis and scavenging ROS (Atrián-Blasco et al., 2017). Studies have shown that upregulation of MT1/2 expression in ALS model mice (SOD1 transgenic mice) reduces lipid peroxide levels, indicating a role of MT1/2 in oxidative damage (Tokuda et al., 2007). Additionally, MT3 expression in spinal motor neurons has been shown to confer a protective effect on motor neurons in ALS model mice (SOD1-G93A transgenic mice), thereby prolonging their lifespan (Hashimoto et al., 2011). These findings suggest a close association between MTs and the pathological mechanisms of ALS.

#### 5.2.4 Huntington’s disease

HD is an autosomal dominant neurodegenerative disorder caused by an abnormal expansion of the cytosine-adenine-guanine (CAG) trinucleotide repeats in exon 1 of the huntingtin (HTT) gene (Bates, 2003). This mutation results in the production of mHTT, characterized by aberrant folding and aggregation of polyglutamine (polyQ) tracts, thereby contributing to neurotoxicity (Tabrizi et al., 2019). Studies have suggested that the soluble N-terminal fragment (N171) of mHTT may represent a key toxic species involved in HD pathogenesis (Ratovitski et al., 2009).

Accumulating data reveals that Cu plays a vital regulatory role in the pathological course of HD (Fox et al., 2007; Hands et al., 2010; Pérez et al., 1996). Current findings reveal heterogeneity in cerebral Cu metabolism among HD patients; higher levels of Cu have been found in the brains of HD patients in some tests, whereas others observe unchanged or even decreased Cu concentrations (Grubman et al., 2014; Scholefield et al., 2020). Studies have shown that Cu can interact with the metal-binding sites on mHTT monomers and promote the aggregation of mutant huntingtin protein in R6/2 HD mice, thereby driving disease progression (Fox et al., 2007). Moreover, Cu enhances mHTT cytotoxicity in a dose-dependent manner (Lobato et al., 2024). Previous studies have confirmed that Cu^2+^ specifically interacts with the polyQ-containing N171 fragment of HTT and induces its oxidative modification (Fox et al., 2007; Fox et al., 2011).

Notably, Cu can specifically inhibit lactate dehydrogenase (LDH) activity in HD, thereby regulating lactate metabolic homeostasis and affecting the energy substrate supply to neurons (Pamp et al., 2005). In HD transgenic model mice (R6/2 HD mice), abnormal elevation of brain lactate levels accompanied by decreased LDH activity has been observed (Fox et al., 2007). Further studies revealed that Cu disrupts lactate metabolism in the HD brain by inhibiting LDH (Fox et al., 2007). Additionally, Cu exposure significantly upregulates the release of pro-inflammatory cytokines such as TNF-*α*, IL-1β, and IL-4 in both the peripheral and CNS. Clinical measurements have confirmed that the levels of these inflammatory mediators are markedly elevated in the peripheral blood of HD patients (Björkqvist et al., 2008).

#### 5.2.5 Wilson’s disease

WD is an autosomal recessive genetic disorder of Cu metabolism caused by mutations in the ATP7B gene, leading in decreased Cu excretion (Członkowska et al., 2018). In WD, ATP7B dysfunction leads to hypoceruloplasminemia and inadequate hepatic Cu excretion, resulting in Cu buildup in the liver and ensuing hepatic damage (Członkowska et al., 2018). The accumulated Cu is released into the bloodstream predominantly as non-ceruloplasmin-bound Cu (NCC), which deposits in other tissues-particularly the brain-resulting in tissue damage (Bandmann et al., 2015).

Previous studies on the pathogenic mechanisms of WD have predominantly focused on the liver, which is consistent with the primary expression of the ATP7B gene in hepatic tissue. However, CNS involvement in WD is also clinically significant, Cu amounts in the livers of people with WD are said to be about 25 times higher than those in healthy controls (Lech et al., 2007). Moreover, brain Cu concentrations in WD patients can increase by 10 to 15-fold, postmortem analyses have confirmed a correlation between cerebral Cu accumulation and the severity of brain tissue damage in these patients (Mikol et al., 2005; Smolinski et al., 2019). In untreated WD, free Cu levels are significantly elevated, often reaching approximately 50 μg/dL (7.7 μM), and this increase in free Cu is considered the primary driver of Cu-induced toxicity (Brewer et al., 2009). Cu-induced toxicity can lead to pathological alterations in affected tissues. As the most sensitive target of Cu toxicity, mitochondria exhibit morphological changes that are considered early features of WD, comprising augmented electron density, segregation of the inner and outer membranes, giant mitochondria, and several types of ofinclusions (Zischka and Lichtmannegger, 2014). Additionally, alterations in the serum Cu isotope ratio (65Cu/63Cu) in WD patients differ significantly from those in healthy individuals, serving as a possible biomarker for disease progression and a predictive indicator of patient prognosis (Lamboux et al., 2020).

Using a WD model mice (ATP7B−/− mice), Tsvetkov et al. provided *in vivo* evidence for the occurrence of cuproptosis (Tsvetkov et al., 2022). By comparing the livers of aged ATP7B-deficient mice with those of heterozygous and wild-type controls, they observed a loss of lipoylated and Fe-S cluster proteins, along with an increase in Hsp70 abundance (Tsvetkov et al., 2022). Excess Cu promoted the aggregation of lipoylated proteins and destabilization of Fe–S cluster proteins, generating proteotoxic stress and eventually resulting in cell death.

#### 5.2.6 Menkes disease

MD is a rare but severe X-linked recessive disorder of Cu metabolism (Tümer and Møller, 2010). The condition arises from mutations in the ATP7A gene, resulting in impaired Cu absorption and use, which causes systemic Cu shortage and disrupts several physiological processes reliant on Cu-dependent enzymes (Tümer and Møller, 2010).

When ATP7A function is impaired, Cu transport between the intestinal epithelium and the BBB is disturbed, preventing its systemic distribution via the circulatory system and resulting in widespread Cu deficiency (Kaler, 1998). In patients with MD, Cu distribution is markedly uneven: it accumulates in the intestine and kidneys, while Cu levels in the serum, liver, and brain are significantly reduced (Giampietro et al., 2018). Observations from both animal models and MD patients reveal that Cu shortage leads to reduced activity of various Cu-dependent enzymes, including CCO, tyrosinase, and lysyl oxidase, eventually leading in altered physiological functioning (Tümer, 2013).

## 6 Potential therapeutic strategies

The occurrence and development of neurodegenerative diseases are closely related to Cu dyshomeostasis and the induced “cuproptosis.” Restoring brain Cu homeostasis and inhibiting cell death are highly promising therapeutic directions. Metal-protein attenuating compounds (MPACs), Cu chelators, and Cu ionophores have demonstrated considerable potential in the management of neurodegenerative diseases (Table 2) and are frequently applied in both clinical and experimental settings. The following sections provide a detailed overview.

**Table 2:** Potential therapeutic strategies.

Therapeutic strategies	Model/topic type	Mechanism of action	Ref
CQ	AD transgenic mice	Inhibit brain Aβ accumulation	Cherny et al. (2001)
CQ	Transgenic Huntington’s mice	Selectively reduces polyQ levels and inhibits the in vivo accumulation of aggregated HTT.	Nguyen et al. (2005)
PBT2	Patients with AD	Reduce Aβ levels in the CSF.	Lannfelt et al. (2008)
PBT2	*Caenorhabditis elegans* model of HD	Alleviate the toxic effects of polyQ aggregation.	Cherny et al. (2012)
PBT2	Patients with HD	It reduces the Cu-dependent conversion of mHTT monomers into toxic oligomers.	Huntington Study Group Reach2HD Investigators (2015)
PBT434	Animal models of PD	Prevent α-syn accumulation.	Finkelstein et al. (2017)
DPA	Patients with AD	Attenuates oxidative stress-induced damage.	Squitti et al. (2002)
DPA	Patients with WD	Promotes urinary Cu excretion and induces the activation of MTs in patients with WD.	Roberts and Schilsky (2008)
Trientine	Patients with WD	Promotes urinary Cu excretion.	Schilsky et al. (2022)
Trientine	Transgenic mouse models of ALS	Reduce SOD1 aggregation and oxidative damage	Andreassen et al. (2001)
TTM	AβPP/PS1 transgenic mice	Reduces the formation of Aβ plaques in the cortex.	Wang et al. (2018)
TTM	Mouse model of ALS	Reduces spinal cord Cu levels, inhibits lipid peroxidation, and significantly suppresses SOD1 enzymatic activity.	Tokuda et al. (2008)
WTX101	Patients with WD	Enhances biliary Cu excretion and reduces plasma NCC levels.	Weiss et al. (2017)
TDMQ20	Mouse model of AD	Modulates Cu homeostasis and inhibits Cu–Aβ complex-catalyzed oxidative stress.	Sun et al. (2022)
ES	Mottled-brindled mouse	Increase the level of CCO in the brain	Guthrie et al. (2020)
DSF	Mouse model of AD	Upregulates ADAM10 expression and prevents Aβ plaque aggregation.	Reinhardt et al. (2018)
Cu (II) (atsm)	SOD1^G37R^ mouse model of ALS	Increase SOD1 levels	Roberts et al. (2014)
Cu (II) (atsm)	SOD1^G93A^ mouse model of ALS	Reduce oxidative and nitrosative damage in SOD1^G93A^ mice, inhibited the formation of aberrantly phosphorylated and fragmented TAR DNA-binding protein-43 (TDP-43) in the spinal cord.	Soon et al. (2011)
Cu (II) (atsm)	Animal models of PD	Ameliorates Cu metabolic dysregulation, improves dopamine metabolism, and inhibits α-syn oligomerization.	Hung et al. (2012)

CQ, Clioquinol; DPA, D-penicillamine; TTM, tetrathiomolybdate; WTX101, Bis-choline tetrathiomolybdate; TDMQ20, tetradentate monoquinoline 20; AD, Alzheimer’s disease; HD, Huntington’s disease; PD, Parkinson’s disease; WD, Wilson’s disease; ALS, amyotrophic lateral sclerosis; Aβ, amyloid β-protein; polyQ, polyglutamine; HTT, huntingtin; CSF, cerebrospinal fluid; mHTT, mutant huntingtin protein; α-syn, α-synuclein; NCC, non-ceruloplasmin-bound Cu; SOD1, superoxide dismutase 1; ES, elesclomol; DSF, disulfiram; Cu (II)(atsm), Cu (II) complex of diacetylbis(4-methylthiosemicarbazone); AD, Alzheimer’s disease; ALS, amyotrophic lateral sclerosis; CCO, Cytochrome c oxidase; α-syn, α-synuclein.

### 6.1 Cu chelators metal-protein attenuating compounds

MPACs are a class of multifunctional compounds that, unlike conventional chelators, exhibit moderate affinity for bound metal ions and demonstrate mild chelating activity (Fasae et al., 2021). Clioquinol (CQ) is a mild metal chelator capable of crossing the BBB, with moderate affinity for both Cu and zinc (Rivera-Mancía et al., 2010). *In vitro* studies have demonstrated that CQ can reverse Cu^2+^- and Zn^2+^-induced Aβ aggregation and dissolve Aβ deposits in postmortem brain tissues affected by AD at low concentrations (<400 nM) (Cherny et al., 2001) (Table 2). Furthermore, research has shown that CQ diminishes the accumulation of polyQ-expanded proteins and increases cell survival in *in vitro* models of HD (Nguyen et al., 2005) (Table 2). PBT2, a second-generation 8-hydroxyquinoline derivative, was structurally optimized based on CQ (Adlard et al., 2008). A small Phase II clinical trial reported that PBT2 elicited a signal of cognitive improvement in patients with mild AD and reduced CSF Aβ levels (Lannfelt et al., 2008) (Table 2). Moreover, PBT2 improved motor performance in the R6/2 mouse model of HD (a transgenic HD mouse model characterized by early onset, rapid disease progression, and severe symptoms). Similarly, in a *Caenorhabditis elegans* model of HD, PBT2 alleviated polyQ aggregation-associated toxicity and prolonged lifespan (Cherny et al., 2012) (Table 2). Clinical studies also demonstrated that PBT2 reduced the Cu-dependent conversion of mHTT into toxic oligomers (Huntington Study Group Reach2HD Investigators, 2015) (Table 2). In addition, PBT434 is a novel quinazolinone compound with high affinity for Cu and is currently under investigation for the treatment of PD. Studies have shown that PBT434 effectively prevents *α*-synuclein accumulation and improves motor function in PD animal models (hA53T α-synuclein transgenic mice) (Finkelstein et al., 2017) (Table 2).

### 6.2 Cu chelators

Cu chelators are commonly used to facilitate the removal of excess Cu from the body. D-penicillamine (DPA) is a Cu chelator with high affinity for Cu ions but limited ability to penetrate the BBB (Squitti et al., 2002). While DPA has been found to decrease oxidative stress in people with AD, it does not impact the clinical course of the condition (Squitti et al., 2002) (Table 2). DPA is also one of the most often utilized Cu chelators in the treatment of WD (Roberts and Schilsky, 2008). It can promote the excretion of Cu into urine and induce the action of MT in WD patients to treat the disease (Roberts and Schilsky, 2008) (Table 2). In the treatment of WD, trientine exhibits a Cu-lowering efficacy comparable to that of DPA, although with a reduced risk of neurological decline, making it the preferable option for individuals intolerant to DPA (Schilsky et al., 2022) (Table 2). Research has found that trientine has a significant ameliorative effect on the progression of ALS in transgenic mice (G93A mice), increasing survival rates (Andreassen et al., 2001) (Table 2). Tetrathiomolybdate (TTM) has demonstrated significant therapeutic effects in the treatment of WD (Brewer, 2003). TTM inhibits Cu absorption, presents a lower risk of early neurological damage, and exhibits a rapid onset of action (Brewer, 2003). Administration of TTM to AβPP/PS1 transgenic mice significantly reduced the formation of cortical Aβ plaques and improved learning ability (Wang et al., 2018) (Table 2). Furthermore, studies in ALS model mice (SOD1-G93A mice) have shown that TTM effectively reduces spinal cord Cu levels, inhibits lipid peroxidation, and markedly suppresses SOD1 enzymatic activity, thereby delaying disease onset and extending survival (Tokuda et al., 2008) (Table 2). Notably, TTM is considered highly unstable under conventional use and is ineffective when used as a single agent. Bis-choline TTM (WTX101) is an oral, first-in-class Cu-binding chemical that exhibits greater stability than TTM (Yu et al., 2006). It enhances biliary Cu excretion and reduces plasma NCC levels in patients with WD (Weiss et al., 2017) (Table 2). The specific Cu chelator tetradentate monoquinoline 20 (TDMQ20) acts on the cholinergic system to regulate Cu homeostasis in the brains of AD patients. It inhibits Cu–Aβ complex–catalyzed harmful oxidative stress, thereby improving cognitive and behavioral performance in AD model mice (5xFAD mice) (Sun et al., 2022) (Table 2).

### 6.3 Cu ionophores

Cu ionophores exhibit unique mechanisms of action and therapeutic potential in neurodegenerative diseases. ES, functioning as a Cu ionophores, forms complexes with Cu and transports them into mitochondria, thereby targeting mitochondrial function (Tarin et al., 2023). Studies in mouse model of MD (Mo-br mice) have shown that ES can transport Cu ions into mitochondria, elevate brain CCO levels, prevent detrimental neurodegenerative changes, and improve survival (Guthrie et al., 2020) (Table 2). ADAM10 is a metalloprotease whose elevated levels in AD mouse models (single-transgenic APP[V717I] mice) appear to alleviate Aβ plaque formation as well as learning and memory impairments (Postina et al., 2004). Acute treatment with DSF in AD model mice (5xFAD mice) induced ADAM10 expression in peripheral blood cells, decreased plaque accumulation in the dentate gyrus, and ameliorated behavioral impairments (Reinhardt et al., 2018) (Table 2).

### 6.4 Others

The Cu (II) complex of diacetylbis (4-methylthiosemicarbazone) [Cu(II) (atsm)] has been described as an effective therapeutic strategy for ALS and has been validated in several ALS mouse models (SOD1G37R and SOD1G93A). Studies have shown that the Cu in Cu (II) (atsm) is transferred to mutant SOD1, increasing its Cu content and thereby raising the overall level of fully metallated (holo) SOD1. This significantly ameliorates motor dysfunction and improves survival in SOD1G37R mice, indicating that increasing SOD1 metal content represents an effective therapeutic strategy for SOD1-linked ALS (Roberts et al., 2014) (Table 2). Nitrosative damage refers to the covalent modification of biomolecules by excessive nitric oxide and its reactive derivatives, particularly peroxynitrite, which disrupts their normal structure and function, ultimately leading to cellular dysfunction or death (Nakamura and Lipton, 2011). Cu (II)(atsm) is considered an anti-nitrosative agent (Gil-Bea et al., 2017). Oral administration of Cu (II)(atsm) in SOD1G93A mice reduces oxidative and nitrosative damage and prevents the accumulation of abnormally phosphorylated and fragmented TAR DNA-binding protein-43 (TDP-43) in the spinal cord, a key protein involved in ALS progression (Soon et al., 2011) (Table 2). Notably, Cu (II)(atsm) also exhibits neuroprotective effects in various PD animal models (MPTP-lesioned C57BL/6 mice, 6-OHDA-lesioned mice, hA53T *α*-synuclein transgenic mice, and MPTP-lesioned hA53T transgenic mice). It improves disrupted Cu metabolism, alleviates PD-induced motor and cognitive deficits, enhances dopamine metabolism, and inhibits α-synuclein aggregation (Hung et al., 2012) (Table 2).

## 7 Future directions

**Figure 6:**
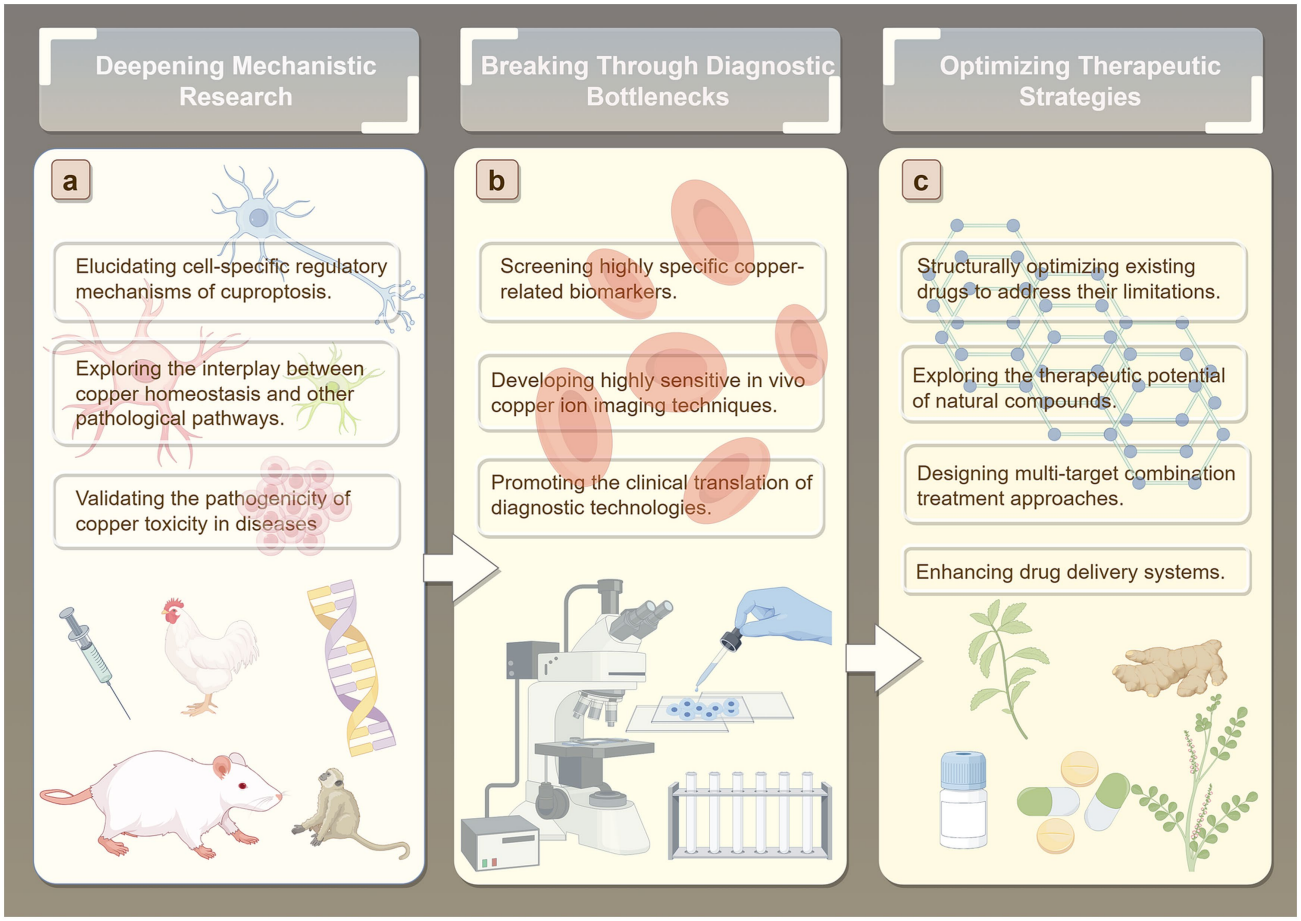
Future directions. **(A)** Deepening Mechanistic Research. **(B)** Breaking Through Diagnostic Bottlenecks. **(C)** Optimizing Therapeutic Strategies.

Over the past few decades, average life expectancy has steadily increased, leading to a global rise in the incidence of age-related neurodegenerative diseases, which imposes a substantial burden on both society and patients (Wang et al., 2024). Accumulating evidence suggests that one potential risk factor for neurodegenerative diseases is age-associated dysregulation of metal homeostasis (Zhong et al., 2024; Zhu et al., 2024; Wang et al., 2025; Chen et al., 2025). Although extensive research has established the central role of Cu dyshomeostasis and cuproptosis in the onset and progression of neurodegenerative diseases, numerous scientific questions and technical challenges in this field remain unresolved (Yang et al., 2024) Therefore, we propose that future studies can be systematically advanced in several key directions to identify novel targets for early intervention and precision therapy of neurodegenerative diseases (Figure 6).

### 7.1 Deepening mechanistic research

Regarding potential underlying mechanisms, existing studies have established a close association between Cu toxicity and pathological processes such as mitochondrial dysfunction and oxidative stress (Tsvetkov et al., 2022). However, the precise molecular mechanisms by which Cu dyshomeostasis triggers neuronal cuproptosis, the shared and disease-specific features of cuproptosis regulatory pathways across neurodegenerative diseases such as AD and PD, and the dynamic regulation of Cu chaperones including ATP7A/B and CTR1 during disease progression remain largely unclear. Future research may elucidate the cell-specific regulatory mechanisms underlying copper-induced cell death, focusing on core brain cell types such as neurons, astrocytes, and microglia. This will involve identifying differences in copper ion transport, storage, and metabolic pathways across distinct cell types, revealing how copper homeostasis imbalances influence cellular susceptibility to copper-induced death. Particular emphasis will be placed on investigating the dynamic distribution and functional effects of copper ions within key subcellular structures, including the synaptic cleft, mitochondria, and lysosomes. In addition, investigating the crosstalk between Cu homeostasis and other pathological pathways will be crucial. Specifically, studies should explore the interactions between Cu dyshomeostasis and core pathological events such as Aβ, tau, and α-synuclein aggregation, neuroinflammation, and autophagy dysfunction, to determine whether cuproptosis serves as a central node connecting these pathological processes. Moreover, the pathogenic relevance of Cu toxicity should be validated using conditional knockout models of Cu transport proteins and complementary *in vitro* and *in vivo* functional assays. This approach would clarify the causal role of Cu toxicity in specific neurodegenerative diseases and provide direct experimental evidence to support the hypothesis that “blocking Cu toxicity can slow disease progression,” thereby facilitating the identification of novel therapeutic targets.

### 7.2 Breaking through diagnostic bottlenecks

Early diagnosis of neurodegenerative diseases remains a major challenge (Amooei et al., 2023; Lee et al., 2020). On one hand, pathological changes often occur years or even decades before the onset of clinical symptoms, making pre-symptomatic diagnosis difficult with conventional clinical assessments. On the other hand, early-stage symptoms are often nonspecific, easily confounded with other disorders, which leads to misdiagnosis or underdiagnosis. The current biomarker identification system is also incomplete, further limiting the accuracy and reliability of early detection. These factors collectively result in extremely low rates of early diagnosis (Chen et al., 2025). Given the critical role of Cu ions in the onset and progression of NDs, both their concentration and distribution patterns have potential as biomarkers, offering a promising avenue for early diagnosis (Squitti et al., 2009). Future efforts should focus on developing Cu homeostasis-based diagnostic technologies. This could include systematic analysis of easily accessible biological samples, such as cerebrospinal fluid and blood, to quantify Cu ion levels and changes in Cu chaperone proteins, combined with machine learning approaches to construct multi-indicator diagnostic models that enhance early detection accuracy. Improvements in imaging modalities, such as magnetic resonance imaging (MRI) and positron emission tomography (PET), are also needed. Development of molecular probes that specifically bind to free Cu or Cu chaperone proteins in the brain could enable non-invasive, real-time monitoring of cerebral Cu distribution, providing a visual tool for early diagnosis and disease progression assessment. For identified Cu-related biomarker candidates, rapid and convenient detection kits (e.g., immunochromatographic strips or electrochemical sensors) should be developed and validated through multicenter, large-cohort clinical studies. Establishing standardized detection protocols will be essential to translate these techniques from the laboratory into clinical practice.

### 7.3 Optimizing therapeutic strategies

Currently, drugs targeting Cu homeostasis primarily include MPACs, Cu chelators, and Cu ionophores. However, existing therapeutic approaches can only temporarily or partially alleviate symptoms and are largely unable to effectively halt or reverse disease progression. Moreover, these drugs exhibit multiple limitations, such as restricted efficacy, notable side effects, and poor blood–brain barrier (BBB) penetration. For instance, in the mid-20th century, widespread use of clioquinol (CQ) in Japan led to an outbreak of subacute myelo-optic neuropathy (SMON) (Konagaya et al., 2004). CQ can also induce deficiencies in essential trace metals, including Cu, zinc, and iron (Schaumburg and Herskovitz, 2008; Tjälve, 1984). D-penicillamine (DPA) has been associated with immune reactions and nephrotoxicity (Ala et al., 2007), and its therapeutic efficacy is limited due to poor BBB permeability (Brewer, 2005). Tetrathiomolybdate (TTM) also exhibits adverse effects, such as anemia, leukopenia, and elevated transaminase levels (Brewer et al., 2006). Notably, both CQ and PBT2 failed to reach primary endpoints in phase II clinical trials for AD. One potential reason for their failure is that, as MPACs, they may lack sufficient selectivity, failing to specifically target Cu or zinc ions within pathological A*β*-metal aggregates while inadvertently disturbing the function of other essential metalloproteins, thereby limiting efficacy and potentially introducing safety risks (Zhao et al., 2021). Future research could focus on developing highly selective Cu homeostasis modulators and optimizing the structure of existing drugs to overcome their limitations. Natural compounds, which represent a valuable cultural and medicinal heritage (Zhao et al., 2024; Nan et al., 2023), have shown significant potential in preventing Cu-induced neurotoxicity. Systematic screening of natural compounds with Cu-chelating, antioxidant, and neuroprotective activities—such as curcumin, epigallocatechin-3-gallate (EGCG), and rutin—could identify candidates for drug development. Structural modification may enhance their stability and target affinity, while mechanistic studies could elucidate how these compounds regulate Cu homeostasis (e.g., EGCG modulating Aβ-Cu aggregation; rutin mitigating Cu-mediated oxidative neuronal damage), providing a theoretical basis for therapeutic development (Secker et al., 2023; Arowoogun et al., 2021; Abbaoui et al., 2017; Hyung et al., 2013). Furthermore, multi-target combination therapies designed according to the complex pathological mechanisms of neurodegenerative diseases may enhance treatment efficacy. The design and synthesis of hybrid compounds combining natural compounds and metal chelators has emerged as a promising direction. For example, combining the pharmacophore of resveratrol with CQ has yielded a novel multi-target compound (Mao et al., 2014) with good BBB permeability and potent inhibition of Cu-induced β-amyloid aggregation, demonstrating remarkable therapeutic potential (Saini et al., 2019). Additionally, nanoparticle-based drug delivery systems, a rapidly advancing technology, have been applied in various studies to treat different diseases (Xiao et al., 2022; Hernando et al., 2016). Future research could further explore these systems to provide new strategies for treating neurodegenerative diseases.

## 8 Conclusion

Cu is an essential bio-metal that plays a critical role in numerous biological processes in the human body. Systemic Cu homeostasis is dynamically maintained through intestinal absorption, hepatic storage, and circulation. Excess Cu ions not only act as potent promoters of oxidative stress, exacerbating oxidative damage, but can also directly trigger mechanisms associated with cuproptosis. Cuproptosis, a newly characterized, Cu-dependent regulated form of cell death, is distinctively dependent on mitochondrial respiration, Cu-mediated aggregation of lipoylated proteins, and loss of Fe-S cluster proteins. This discovery reveals a novel link between Cu-induced cell death and mitochondrial metabolism, opening potential therapeutic avenues for various diseases, including neurodegenerative disorders. In summary, research on Cu homeostasis and the mechanisms of cuproptosis represents a highly promising field, and elucidating their roles in the pathogenesis and treatment of neurodegenerative diseases is of critical importance.
